# Papillary fibroelastoma of the aortic valve - a case report and literature review

**DOI:** 10.1186/1749-8090-5-84

**Published:** 2010-10-17

**Authors:** Neerod K Jha, Michael Khouri, Donogh M Murphy, Alessandro Salustri, Javed A Khan, Moataz A Saleh, Friederike Von Canal, Norbert Augustin

**Affiliations:** 1 Division of Adult Cardiac Surgery, Institute of Cardiac Sciences, Sheikh Khalifa Medical City (Managed by Cleveland Clinic), PO Box-51900, Abu Dhabi-UAE; 2 Division of Cardiology, Institute of Cardiac Sciences, Sheikh Khalifa Medical City (Managed by Cleveland Clinic), PO Box-51900, Abu Dhabi-UAE; 3 Department of Laboratory Medicine, Sheikh Khalifa Medical City, (Managed by Cleveland Clinic), PO Box-51900, Abu Dhabi-UAE; 4 Department of Anaesthesiology, Sheikh Khalifa Medical City, (Managed by Cleveland Clinic), PO Box-51900, Abu Dhabi-UAE

## Abstract

The prevalence of primary cardiac tumour ranges from 0.0017-0.28% and papillary fibroelastoma is rare but not uncommon benign cardiac neoplasm. Currently, with the advent of higher-resolution imaging technology especially transoesophageal echocardiography such cases being recognized frequently. The clinical presentation of these tumours varies from asymptomatic to severe ischaemic or embolic complications. We herein, present a 50-year-old female patient with a papillary fibroelastoma of the aortic valve arising from the endocardium of the right coronary cusp very close to the commissure between the right and non-coronary cusps. The patient presented with angina-like chest pain and was investigated using echocardiography and CT angiographic modalities in addition to the usual investigations. The differential diagnosis considered was a thrombus, myxoma, Lambl's excrescence and infective vegetation. The surgical management included a prompt resection of the tumour on cardiopulmonary bypass avoiding injury to the aortic valve. The patient recovered well. A review of the literature suggests that the cardiac papillary fibroelastoma is a rare but potentially treatable cause of embolic stroke and other fatal complications, therefore, a strong suspicion; appropriate use of imaging modality, preoperative anticoagulation and urgent surgical resection is warranted. Also, possibility of this diagnosis should be kept in mind while managing cardiac or valvular tumours.

## Introduction

Although the prevalence of primary cardiac tumors ranges from 0.0017-0.28%, the papillary fibroelastomas (PFE) are second most common benign neoplasm of the cardiac valves after myxomas [1-3]. Currently, with the advent of higher- resolution imaging technology such cases are diagnosed more frequently [1-14]. We herein, present a 50-year-old female patient with PFE arising from the endocardium of the right coronary cusp of the aortic valve that presented with recurrent angina-like chest pain and successfully managed with valve-sparing resection of the tumor on cardiopulmonary bypass. This report not only highlights typical presentation of this tumor but also reminds us to keep this possibility in patients who present with mass in the ascending aorta, aortic valve or those associated with angina-like or neurological symptoms.

## Case report

**Figure 1:**
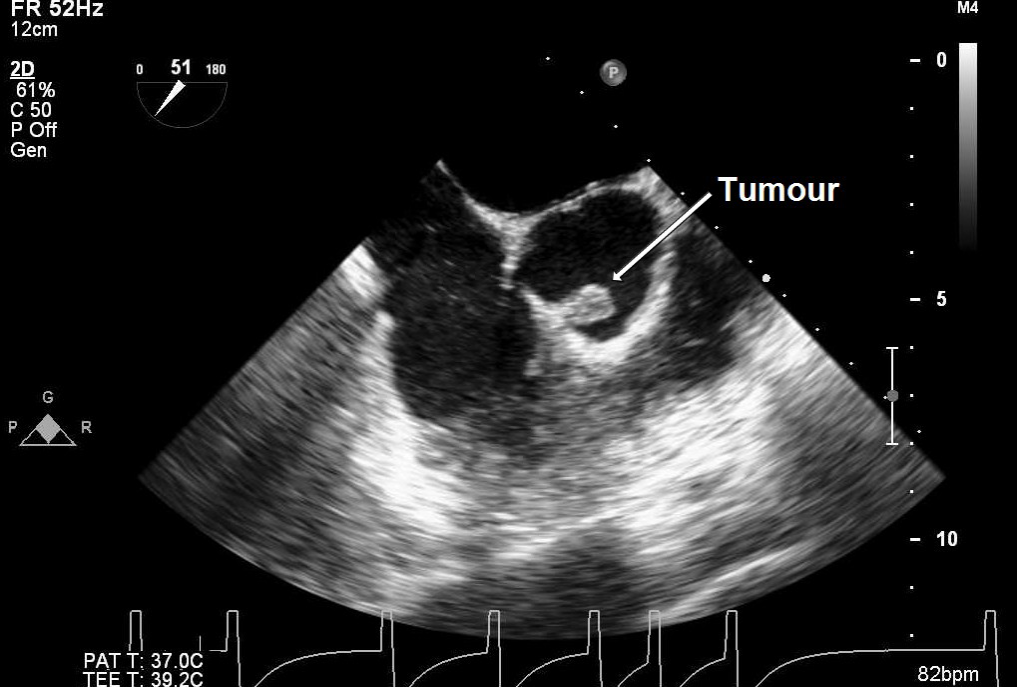
**Trans-oesophageal echocardiography showing a mobile, spherical pedunculated tumour mass of 1.2 × 1 cm in size at the right coronary aortic cusp (ME AV short-axis view)**.

**Figure 2:**
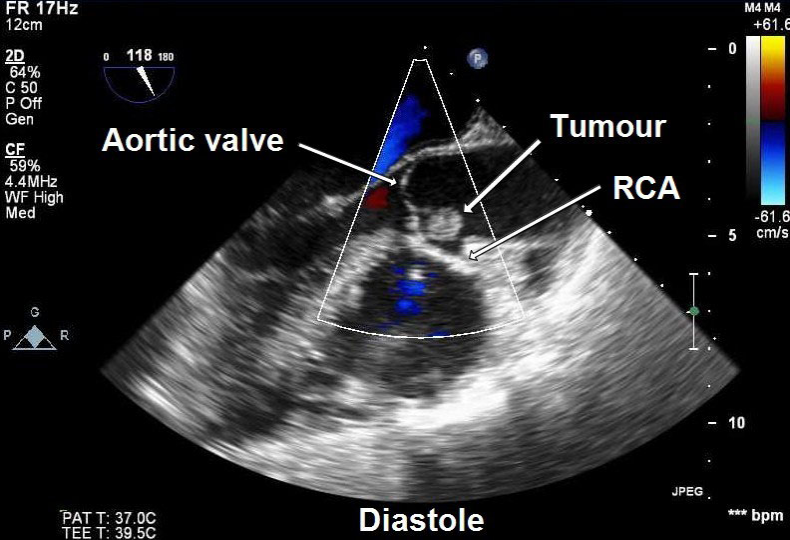
**Trans-oesophageal echocardiography showing supra valvular tumour during diastole near the right coronary artery (RCA) ostium (ME AV long-axis view)**.

**Figure 3:**
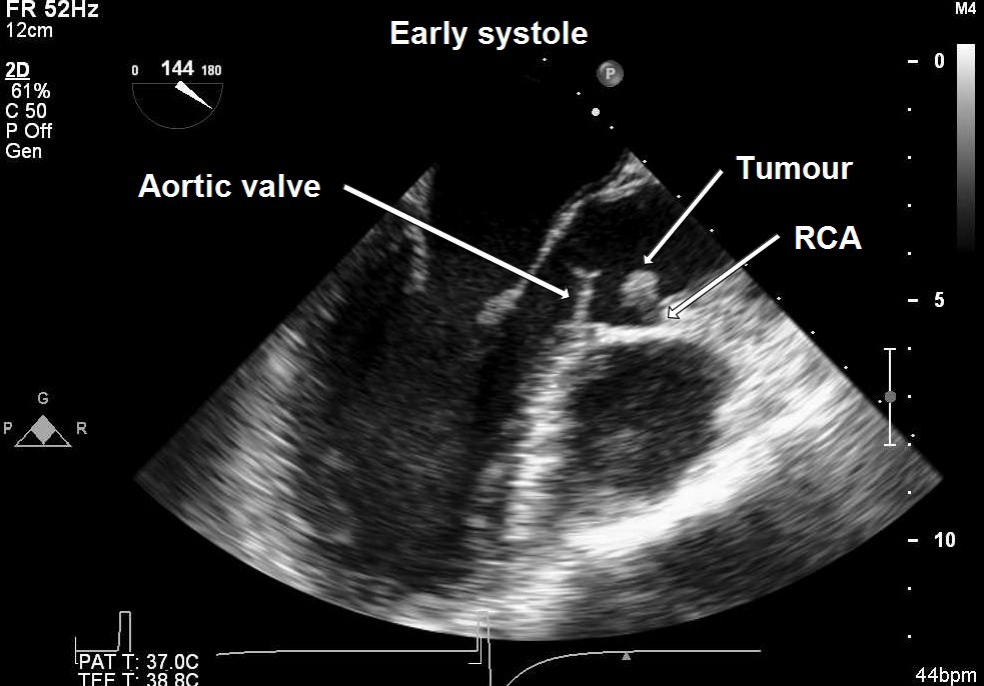
**Trans-oesophageal echocardiography showing supra valvular tumour during early systole moving away from the right coronary artery (RCA) ostium (ME AV long-axis view)**.

**Figure 4:**
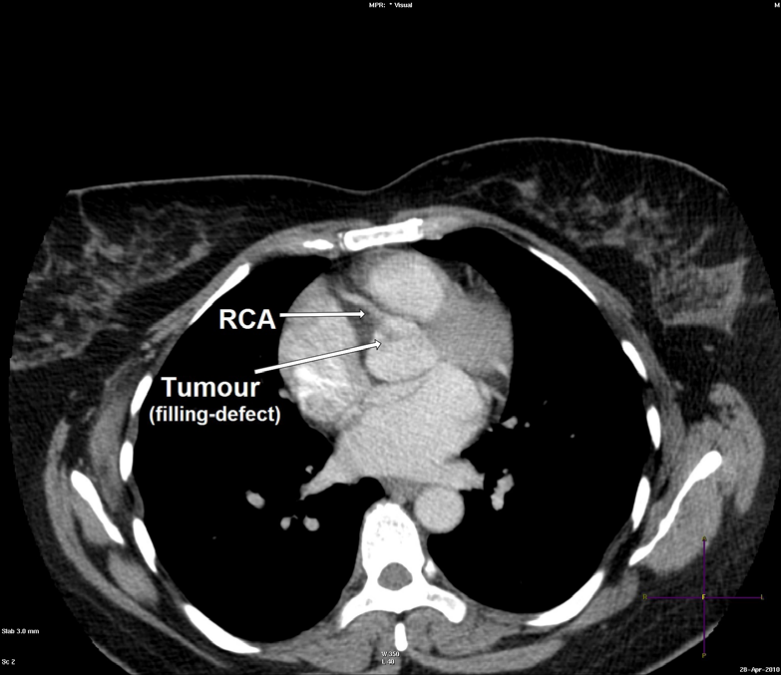
**Contrast computerized tomography image showing a filling-defect (tumour) attached to the aortic valve near the origin of the right coronary artery (RCA)**.

A 50-year-old female presented to our hospital for evaluation of chest pain. She had history of recurrent, vague, central chest pain with radiation to inter-scapular area. The pain was compressive and mild in nature and was not associated with effort. The clinical examination and routine blood laboratory investigations were unremarkable. The electrocardiography including stress test was also inconclusive. The chest x ray was normal. A 2-D and transoesophageal echocardiography (TEE) revealed presence of an echodense supra valvular, pedunculated, spherical mass of 1.2 × 1 cm in size about 1.2 cm above the aortic annulus (Figure 1). This supra valvular echogenic mass was found to be moving and displaced during each phase of the cardiac cycle and it was very close to the orifice of the right coronary artery (RCA) (Figure 2 and 3). However, the aortic valve and other cardiac structures were normal. There was no regurgitation of the aortic valve. A contrast-enhanced computerized tomography scan of the chest confirmed the presence of a mildly ill-defined, non-enhancing, hypodense nodular lesion of approximate size 1.0 × 0.8 × 0.7 cm in the aortic root, just adjacent to the origin of right coronary artery (Figure 4). Based upon the findings as above, a differential diagnosis was made which included, thrombus, myxoma, fibroelastoma and inflammatory mass.

**Figure 5:**
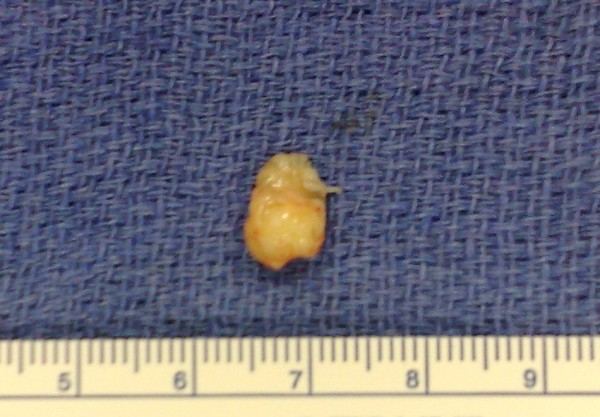
**Gross specimen of resected mass**.

**Figure 6:**
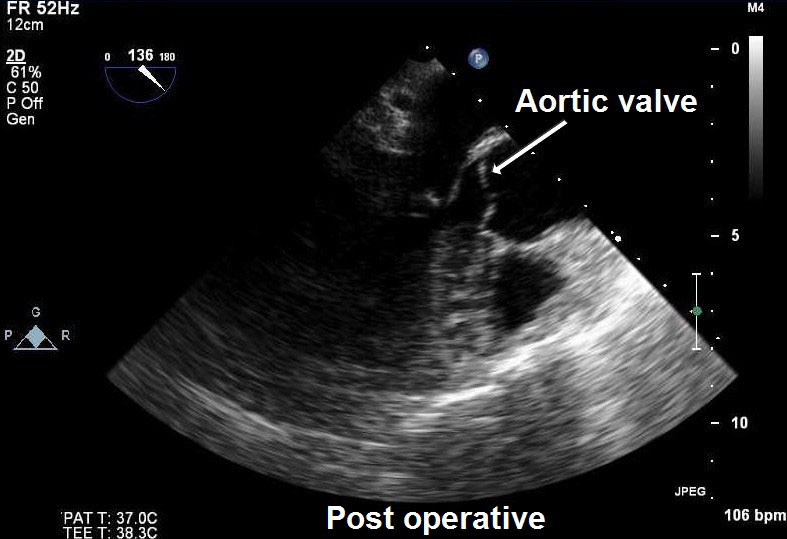
**Postoperative trans-oesophageal echocardiography confirming complete resection of the tumour and normal aortic valve (ME AV long-axis view)**.

In view of the possibility of embolism and unknown nature of the pathology, the patient was taken for urgent surgical resection under standard cardiopulmonary bypass at systemic hypothermia (32 degree Celsius) and systemic heparinisation. The ascending aortic and right atrial cannulation was done before institution of cardiopulmonary bypass. The aortic cross clamp was applied and heart was arrested using antegrade blood cardioplegia via the root. Subsequently, a transverse aortotomy was performed proximal to the aortic root. On exploration a 1 × 1 cm pedunculated tumor mass was found to be attached to the right coronary cusp very close to the commissure between the right and non-coronary cusp of the aortic valve. The tumor mass was firm, glistening, friable and looked filamentous on gross examination (Figure 5). The aortic valve was tri-leaflet and structurally and functionally found to be normal. A complete resection of the tumor was achieved which was confirmed by postoperative TEE (Figure 6). The aortic valve was found to be competent and functionally well without any residual defect or perforation after the procedure. The patient had an uneventful weaning from the cardiopulmonary bypass and recovered well in the immediate post operative period.

**Figure 7:**
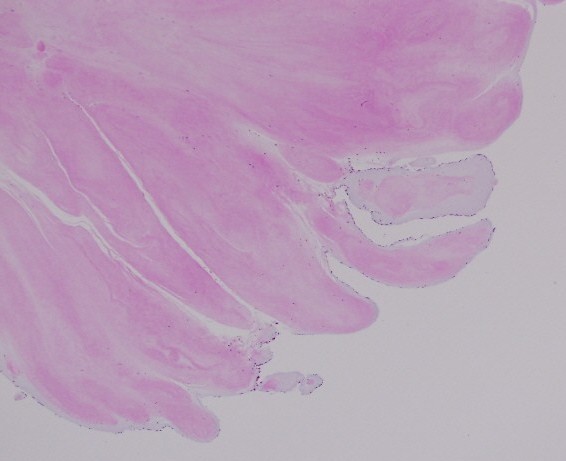
**Histological section of the excised mass showing benign papillary lesion comprised of a single layer of endocardial cells overlies a thin layer of mucopolysaccharide matrix and underlying, almost acellular, avascular stroma composed predominantly of elastic fibers and a small amount of collagen**. (Hematoxylin and Eosin stain, magnification × 40)

Histopathology examination of the resected tumor revealed a papillary proliferation including few fibroblasts, collagen and elastic fibers, covered with hyperplastic endothelial cells. These features confirmed the diagnosis of papillary fibroelastoma (Figure 7). The postoperative course was uneventful and the patient was discharged in a satisfactory condition on 7^th ^day.

## Discussion

Cardiac papillary fibroelastomas are classified as primary benign endocardial tumours arising from the normal component of the endocardium like fibrous tissue, elastic fibers or smooth muscle cells. Characteristically they have a short pedicle and multiple papillary fronds similar to a sea anemone [1]. They often (85%) originate from the valvular endocardium. The aortic valve (29%), mitral valve (25%), tricuspid valve (17%) and pulmonary valves (13%) are involved in that order [1]. However, PFE arising from semilunar valves are located with equal frequencies on the ventricular and arterial sides of the valves. In addition, non-valvular origin was observed in approximately 16% cases that included left and right ventricular septal and mural endocardial surfaces, atrial endocardium, papillary muscles, chordae tendinae or intima of the right coronary ostium [1-10]. In addition, multifocal "tepete" (carpet-like) PFE of the left ventricular cavity has also been reported [2].

The origin of PFE is unclear and various possible causes have been mentioned in the literature. They have been considered as hamartomas, organized thrombi, iatrogenic (post radiation, surgery) or inflammatory foci due to unusual endocardial responses to infection or hemodynamic trauma [3]. However, some authors believe that PFE's are true neoplasms [1-3].

Clinical presentation of PFE's varies from asymptomatic to severe thromboembolic complications, myocardial ischemia, infarction and stroke. However, pulmonary embolism, congestive heart failure, near-syncope, ventricular fibrillation and sudden death have also been reported [1-10]. Embolisation may occur from fragments of the fronds of the tumour or from a thrombus that frequently forms on the tumour 'nidus' due to platelet or fibrin aggregates.

In our patient, the atypical vague chest discomfort and pain could have been due to the partial, intermittent obstruction or limitation of blood flow through the right coronary artery orifice in the aortic root due to the strategic location and variable mobility of the tumour mass during various phases of cardiac cycle (Figure 2 and 3). Therefore, this could be considered as angina-like symptom.

The diagnosis is usually made by 2-dimentional or transoesophageal echocardiography. Recently, 3-D echocardiography, magnetic resonance imaging and multislice spiral computed tomography have also been used for better delineation of similar tumors [6,8]. Typical echocardiographic features include a small (1-4 cm), highly mobile mass with a pedicle attached to the valve or endocardial surface and a frond-like appearance with or without multifocal involvement. The contrast CT image typically shows a filling defect in the aortic root adjacent to the origin of coronary artery [6].

Despite the benign nature of this tumour, it carries very high risk of embolic complications including neurological deficit. The fragile nature and frond-like papillary tissues of the tumour itself is prone to thromboembolism [1-7]. Therefore, once diagnosed, urgent surgical management is indicated even in the asymptomatic patients [1,4,5,7,10]. The management of such tumours also includes early anticoagulation. The surgical management requires extracorporeal circulation and an aortotomy which is similar to that used in typical aortic valve replacement procedures. The PFE's are usually pedunculated and may be easily removed with associated endocardial tissue. Care should be taken to avoid fragmentation of the tumour tissue. Also, aortic valve should be preserved preferably. In case, if there is resultant valve defect, it should be repaired, otherwise a valve replacement is warranted. The surgical resection is curative, safe and well tolerated [5,7,9,10]. Intraoperative TEE is essential to assess valvular function after tumour resection. Re-growth of the tumour after resection has not been reported, and it requires long-term TEE follow-up studies to confirm.

Law 'et al' have done a review of the English language literature using 'Pub Med' (US National Library of Medicine) to identify previously published cases of PFE from 1997 up to 2008 [2,7]. They found 833 cases published so far with a male preponderance (58%) and mean age of 56 years. The tumour size ranged from 2 to 70 mm. The two most preferred locations were aortic (44-52%) and mitral valve (35-40%). In majority of the cases echocardiography was the imaging modality (98%) and excision of the tumour (79%) as preferred treatment. We have tried to update the review of literature including recently published reports (Table 1).

**Table 1:** Previously reported cases of cardiac papillary fibroelastoma

Reference	Number of patient	Mean age (yr)	Sex distribution	Size (mm)	Site	Presentation	Management
**Grinda 'et al'**^**1**^ **(1999)**	04	54	3 Males 1 Female	10	1 MV 1 TV 1 AV	CVA Aphasia Syncope TIA	Excision+MVR Excision+TVR Excision+AVRp Excision

**Saw 'et al'**^**14**^ **(2001)**	03	45	2 Females	6-7	1 IVS 2 MV	Stroke Abdominal pain, VSD	Excision

**Darvishian 'et al'**^**7**^ **(2001)**	02	50	1 Male	10-15	2 MV	Dyspnea Ataxia Dysphasia	Mitral Repair+ excision

**Gowda 'et al'**^**13**^ **(2003)**	725	70	56% Males	2-70	44% AV 35% MV	32% Stroke 13% Angina	81% Excision 10% AVR

**Sato 'et al'**^**9**^ **(2003)**	01	74	Male	9	LVOT	AF Chest discomfort	Excision

**Ngaage 'et al'**^**12**^ **(2005)**	88	62	71% Males	-	52% AV 18%- LVOT	52% -Dyspnea 32% TE	83% excision

**Kumbala 'et al'**^**11**^ **(2008)**	01	60	Male	9	AV	TIA	Excision

**Law' et al'**^**2**^ **(2009)**	01	25	Male	9	LVOT (multiple)	Stroke	Excision+MVRp

**Bicer 'et al'**^**3**^ **(2009)**	01	72	Male	12	LA	Stroke	Excision

**Parthenakis 'et al'**^**8**^ **(2009)**	01	29	Female	12	AV	Asymptomatic	Excision

**Domenech 'et al'**^**4**^ **(2010)**	01	59	Female	11	LV	Stroke	Resection

AV-aortic valve, IVS-interventricular septum, MV-mitral valve, CVA-cerebrovascular accident, TIA-transient ischemic attack, VSD-ventricular septal defect, AF- atrial fibrillation, LVOT-left ventricular outflow tract, LA- left atrium, LV- left ventricle, MVR-mitral valve repair, TVR-tricuspid valve repair, AVRp-aortic valve replacement, MVRp-mitral valve replacement, AVR-aortic valve repair

## Conclusion

Cardiac PFE's are not uncommon tumours and should be considered in the differential diagnosis of cardiac masses. A strong suspicion, appropriate use of imaging modality, pre operative anticoagulation and urgent resection of the tumour is not only life saving but also avoids tumour-related vascular, embolic or neurological complications.

## Competing interests

The authors declare that they have no competing interests.

## Authors' contributions

All authors have contributed in case management, manuscript preparation and image acquisition.

## Consent

Written informed consent was obtained from the patient for publication of this case report and any accompanying image. A copy of the written consent is available for review by the Editor-in-Chief of this journal.
